# Brief Intervention Impact on Truant Youth Attitudes to School and School Behavior Problems: A Longitudinal Study

**DOI:** 10.5539/jedp.v4n1p163

**Published:** 2014-03-12

**Authors:** Richard Dembo, Rhissa Briones-Robinson, Jennifer Wareham, Ken C. Winters, Rocío Ungaro, James Schmeidler

**Affiliations:** 1 Department of Criminology, University of South Florida, Tampa, FL; 2 Department of Psychiatry, University of Minnesota, Minneapolis, MN; 3 Department of Criminal Justice, Wayne State University, Detroit, MI; 4 Mt. Sinai School of Medicine, New York, NY

**Keywords:** truancy, school problems, growth model, Brief Intervention

## Abstract

Truancy continues to be a major problem, affecting most school districts in the U.S. Truancy is related to school dropout, with associated adverse consequences, including unemployment and delinquency. It is important to obtain a more complete picture of truants' educational experience. First, the present study sought to examine the longitudinal growth (increasing/decreasing trend) in truant youths' attitudes toward school and misbehavior in school (disobedience, inappropriate behavior, skipping school). Second, this study focused on examining the impact of a Brief Intervention (BI) targeting the youths’ substance use, as well as socio-demographic and background covariates, on their attitudes toward school and school behavior problems over time. A linear growth model was found to fit the attitudes toward school longitudinal data, suggesting the youths’ attitudes toward school are related across time. An auto-regressive lag model was estimated for each of the school misbehaviors, indicating that, once initiated, youth continued to engage in them. Several socio-demographic covariates effects were found on the youths’ attitudes towards school and school misbehaviors over time. However, no significant, overall BI effects were uncovered. Some statistically significant intervention effects were found at specific follow-up points for some school misbehaviors, but none were significant when applying the Holm procedure taking account of the number of follow-ups. The implications of these findings are discussed.

## 1. Introduction

### 1.1 Introduction to the Problem

Truancy continues to be a major problem in affecting most school districts in the U.S. Generally, truancy is defined as unauthorized, intentional absence from compulsory schooling. Although truancy is hard to quantify, since a uniform definition of truancy is lacking and there is inconsistent reporting and tracking procedures across school districts, (National Center for School Engagement, 2006; U.S. Department of Education, n.d.), it is estimated that thousands of youth are absent from school each day. For example, recent statistics on truancy in Los Angeles County indicate high rates of unexcused absences, with the highest rates being found in urban high schools (Dropout Nation, 2010). Two hundred thousand Los Angeles County students were truant during the 2008-2009 school year, which represented 16 percent of all students attending schools in the county. Fifty-seven of L.A. County's 88 school districts experienced truancy rates greater than 10 percent. Similarly, Colorado truancy data for 2010 to 2011 (Colorado Department of Education, 2011) indicated truancy rates (total student days unexcused absent/total days of possible attendance for all students) above ten percent for many schools, including several Denver area schools. Comparable statistics pointing to the high level of truancy problems can be found in other jurisdictions (Garry, 2001).

### 1.2 Research on Correlates for Truancy

Truancy appears to be an early sign for a trajectory toward more negative behaviors (National Center for School Engagement, 2006). As Garry (2001) observed, truancy may be the beginning of a lifetime of problems among students who routinely skip school. Research has linked truancy to family problems such as family disruption (i.e., divorce) and poor parenting practices (Baker, Sigmon, & Nugent 2001; Dembo et al., in press b; Vaughn, Maynard, Salas-Wright, Perron, & Abdon, 2013; Veenstra, Lindenberg, Tinga, & Ormel, 2010). Youths from families with lower socioeconomic and income are also at greater risk of truancy, than those from higher income families and socioeconomics (Attwood & Croll, 2006; Bell, Rosen, & Dynlacht, 1994; Nolan, Cole, Wroughton, Clayton-Code, & Riffe, 2012; Veenstra et al., 2010). Studies have also indicated certain socio-demographic characteristics of the youth may put him/her at risk of truancy. Youths who are older in age (Hersov & Berg, 1980; Nolan et al., 2012; Vaughn et al., 2013), male (Hersov & Berg, 1980; Veenstra et al., 2010), and of racial or ethnic minority (e.g., Vaughn et al., 2013) are more likely to be involved in truancy.

A respectable amount of research has shown truancy is a risk-factor for various delinquent and criminal behaviors. Truant youth are more involved in alcohol/other drug use than non-truant youth (e.g., Barry, Chaney, & Chaney, 2011; Flaherty, Sutphen, & Ely, 2012; Henry, 2010; Henry, Knight, & Thornberry, 2012; Maynard, Salas-Wright, Vaughn, & Peters, 2012; Vaughn et al., 2013). Of particular interest for the present study, truancy has been positively associated with marijuana use initiation (Henry & Huizinga, 2007; Henry, Thornberry, & Huizinga, 2009) and escalation (Henry & Thornberry, 2010). Truant youth also tend to engage in sexual risk behavior at higher levels than the general adolescent population (Dembo et al., in press a), which may place them at heightened risk for teen pregnancy (Hibbert, Fogelman, & Manor, 1990) and sexually transmitted diseases (STDs), including HIV (Centers for Disease Control and Prevention [CDC], 2009). Truant youth are also more likely to report involvement in delinquent behavior (e.g., Gonzales, Richards, & Seeley, 2002; Henry et al., 2012) and official arrest (Henry et al., 2012; Li et al., 2011).

While there are clearly several covariates of truancy that can impact future problem behaviors, the focus of the present paper is on the attitudes and behaviors of truant youth in school. Truancy is related to poor academic performance and school dropout (Alexander, Entwisle, & Horsey, 1997; Caldas, 1993; Lamdin, 1996; Vaughn et al., 2013), poor attachment to teachers (Attwood & Croll, 2006; de Wit, Karioja, & Rye, 2010; Noguera, 2003; White, 2009), and low educational aspirations (Barry et al., 2011; Veenestra et al., 2010). These issues are exacerbated among minority youth (Brown & Rodriguez, 2009; Noguera, 2003). Truancy is also associated with antisocial and behavioral problems in the school setting, such as fighting, social problems, and rule breaking (Corville-Smith, Ryan, Adams, & Dalicandro, 1998; Kim & Page, 2013; Vaughn et al., 2013). Poor school achievement contributes to adverse socioeconomic consequences, including unemployment and a higher risk of criminal justice involvement (Sprott, Jenkins, & Doob, 2005), with staggering costs to society (Center for Labor Market Studies, 2009; Reschly & Christenson, 2006). Hence, understanding truant youths’ feelings about school and their behavioral issues in school are important in obtaining a more complete picture of their educational experience. Such is the purpose of the present study, involving substance using truant youth in a NIDA-funded Brief Intervention (BI) project.

### 1.3 Purpose and Hypotheses

The present study examines the growth, or temporal increase/decrease, in school-related attitudes and problems among youths identified as truant and the influence of a drug-use intervention for truant youth on this growth. The effect of the intervention on the mean rates of school-related attitudes and problems were examined across five waves of data for the truant youth, controlling for key baseline covariates such as socio-demographics, marijuana use, sexual risk behaviors, and delinquency. As described in more detail in section 2, youth were randomly assigned to either one of two Brief Intervention (BI) conditions for drug-use intervention [two sessions with the youth (BI-Y) or two sessions with the youth and one session with the parent (BI-YP)], or to a Standard Truancy Services (STS). In general, it was expected that the youth would experience increased positive attitudes toward school and decreased behavioral problems in school over time, with youths participating in the BI, especially the BI-YP, demonstrating greater increases in positive attitudes toward school and greater decreases in problem behaviors in school, compared to youths assigned to STS.

Specifically, four hypotheses were tested in this study:

H1: Controlling for socio-demographic and background covariates, youths receiving BI intervention services (BI-Y, BI-YP) will show a greater increase in favorable attitudes toward school over time, than youth receiving STS services.
H2: Controlling for socio-demographic and background covariates, youths receiving BI intervention services (BI-Y, BI-YP) will show a greater reduction in problem behavior in school, than youth receiving STS services.
H3: Controlling for socio-demographic and background covariates, youths receiving BI-YP intervention services will show a greater increase in favorable attitudes toward school over time, than youth receiving BI-Y services.
H4: Controlling for demographic and background covariates, youths receiving BI-YP intervention services will show a greater reduction in problem behavior in school, than youth receiving BI-Y services.

The hypothesized effect of BI-YP services is premised on epidemiological and clinical studies indicating that parent monitoring and support, addressed in the BI-YP session, are associated with reduced risk behavior (e.g., Clark, Kirisci, & Tarter, 1998; Gorman-Smith, Tolan, Loeber, & Henry, 1998; Liddle & Hogue, 2001; Waldron, 1997).

We first discuss the methods used for participant selection and measurement. Next, we discuss the results of our analyses in regard to the objectives and hypotheses informing our study. Finally, we consider the service delivery implications of the findings.

## 2. Methods

Baseline and follow-up interviews were conducted by trained research staff, and all study procedures were approved and monitored for ethics by the university Institutional Review Board (IRB). The main place of recruitment into the study was a Truancy Intake Center (TIC) located at the Hillsborough County Juvenile Assessment Center in Florida. In addition, eligible participants were recruited from a community diversion program and referrals from any Hillsborough County School District (HCSD) social worker or guidance counselor who knew of eligible youth. Project enrollment proceeded as follows. A project staff member met with the youth and his/her parent/guardian, and provided an overview of the project and its services. Eligible participants were informed that project services were free, voluntary, and provided in-home. For interested participants, an in-home meeting was scheduled to discuss the project further, to answer any questions they had, complete the consent and assent processes, and to conduct separate baseline interviews with the youth and his/her parent/ guardian.

Following the completion of the consent and assent processes and baseline interviews, the youth and parent/guardian were randomly assigned to one of three project service conditions: (1) BI-Youth (BI-Y), (2) BI-Youth plus Parent (BI-YP), or (3) Standard Truancy Services (STS). Random assignment was implemented using a method to ensure assignment was balanced, where a randomly generated table of three digits (1, 2, and 3) was used for group assignment and every 12 cases had an equal number assigned to the three groups. Assessment data were collected at baseline and at four follow-up periods: the first three months after the intervention (referred to as 3-month follow-up period); the second three months, or months four through six after the intervention (referred to as 6-month follow-up period); months seven through twelve after the intervention (referred to as 12-month follow-up period); and months 13 through 18 after the intervention (referred to as 18-month follow-up period).

### 2.1 Participants

Eligible youths met the following criteria: (1) age 11 to 17, (2) no official record of delinquency or up to two misdemeanor arrests, (3) some indication of alcohol or other drug use, as determined, for example, by a screening instrument (Personal Experience Screening Questionnaire [PESQ, Winters, 1992]) or as reported by a HCSD social worker located at the TIC, and (4) lived within a 25-mile radius of the TIC. The total sample consisted of 300 youths, who were enrolled and completed baseline interviews between March 2, 2007 and June 22, 2012.

### 2.2 Interventions

#### 2.2.1 Brief Interventions

The goal of the BI sessions was to promote abstinence and prevent relapse in drug use among drug using, truant adolescents. As indicated in section 2.1, participants had to indication alcohol or drug use to be eligible for the study. Adapted from previous work using brief intervention on drug-abusing youth (Winters & Leitten, 2001), the BI incorporated elements of Motivational Interviewing (MI), Rational-Emotive Therapy (RET) and Problem-Solving Therapy (PST) to develop adaptive beliefs and problem-solving skills. Drug involvement was viewed as learned behavior that develops within a context of personal, environmental, and social factors (Catalano, Hawkins, Wells, & Miller, 1991; Clark & Winters, 2002) that shape and define drug use attitudes and behaviors. Maladaptive beliefs and problem-solving skill deficits, developed over the course of an adolescent's learning history and prior experience with drugs, were viewed as primary determinants of drug use. The goal of the BI therapy was to diminish factors contributing to drug use (e.g., maladaptive beliefs) and promote factors that protect against relapse (e.g., problem solving skills) (Winters, Fahnhorst, Botzet, Lee, & Lalone, 2012; Winters & Leitten, 2007). Prior to providing BI services, the BI counselor received training on the treatment manual, personal training from a skilled trainer on all intervention components, and provided BI services to several practice cases with a focus on developing therapist adherence (aided by a rating checklist) and competence (e.g., perceived warmth and interest in the client, presentation clarity, ability to elicit client feedback). With youth and parent/guardian permission, the BI sessions were tape recorded for ongoing fidelity/adherence assessment.

Youths randomly assigned to the BI-Youth (BI-Y) condition were administered two BI sessions with only the youth; youths randomly assigned to the BI-Youth plus Parent (BI-YP) condition were administered the same two BI sessions, and their parents/guardians were administered a separate BI session that involved just the parent. Each BI session lasted for 75 minutes, and the sessions occurred about a week apart. The first youth BI session focused on discussing his/her substance use and related consequences, the level of willingness to change, the causes and benefits of change, and what goals for change the youth wanted to select and pursue. The youth was encouraged to pursue goals of drug abstinence or reduction in drug use. In the second youth session, the counselor reviewed the youth's progress with the agreed upon goals, identified risk situations associated with difficulty in achieving goals, discussed strategies to overcome barriers toward goal achievement, reviewed where the youth was in the process of change, and negotiated either continuation or advancement of goals. The parent BI session addressed the youth's substance use issues, parent attitudes and behaviors regarding this use, parent monitoring and supervision to promote progress towards their child's intervention goals, and parent communication skills to enhance youth-parent connectedness.

##### 2.2.1.1 Ongoing Assessment for Fidelity/Adherence

The BI interventionists successfully completed training in the intervention provided by the project clinical supervisor. To ensure that treatment-as-described was treatment-as-provided, integrity of the intervention was assessed on an ongoing basis. Intervention sessions were audio taped, with parent and youth permission. A counselor adherence checklist was applied to assess use of intervention procedures. The checklist included a list of the content and strategies called for in these sessions and trained coders checked these strategies and procedures were implemented. The obtained ratings of the counselors’ integrity to the intervention were closely monitored in order to trouble-shoot counselor drift. Very few instances of drift were found; in these cases, the interventionists received booster clinical support to address this issue.

#### 2.2.2 Standard Truancy Services (STS)

In addition to the normal truancy services provided by the HCSD, STS youths/families received a referral service overlay of three weekly hour-long visits by a project staff member. Referral assistance provided truant youth and their families in the control condition with an additional resource that is not routinely available to them, and also controlled for service exposure. On each contact occasion, the staff member carried a copy of a Hillsborough County government-developed agency and service resource guide. The agency guide contained hundreds of agency listings, contact persons and telephone/e-mail information. During the STS visits, if the participants indicated they needed assistance finding resources in the community, the project staff member would search through the agency guide and provide the recommended contact information to the participants. For example, if a youth's parents indicated to the staff that they needed help getting financial assistance for food, the staff member would look up the name, address, and phone number of the local agency that could assist them to find such assistance, and give this information to the parents. In addition to inquiring about events since the last session, the visiting staff member asked the youth and his/her parents/guardians: (1) if they used any of the referral services provided to them by staff and (2) if there were any additional service needs–and, if so, provided appropriate referral information. No form of counseling or therapy was offered in the STS condition.

### 2.3 Interview Procedures

Each youth and parent/guardian was paid $15 for completing the in-home, baseline interview. The baseline interviews for parents/guardians averaged 30 minutes; the youth interviews averaged one hour. The 3-month follow-up interview was scheduled for 90 days from the date of the youth's last participation in project services (i.e., the last intervention or STS session).

Some follow-up interviews were not performed at their scheduled time. On 16 occasions, a retrospective interview was performed at the same time as the following interview (e.g., if a 6-month interview was not performed approximately three months after the 3-month interview, two interviews were performed approximately nine months after the 3-month interview). Also, youth who began participation early in the project completed all four follow-up interviews, whereas youth who enrolled at the end of the enrollment period were eligible for only two follow-up interviews (as a result of the fact that the length of the study was not long enough for these late enrollees to complete all follow-up assessments). Overall completion rates of 94.0%, 93.7%, 92.1%, and 88.5% were achieved for the 3-month, 6-month, 12-month, and 18-month follow-up interviews, respectively. Of the completed follow-up interviews, 95.4% of the 3-month, 95.0% of the 6-month, 96.3% of the 12-month, and 99.1% of the 18-month interviews were completed within 60 days of the scheduled interview date. Each youth and parent/guardian was paid $15.00 for each follow-up interview. Most youths were interviewed in their homes; at each follow-up time point, fewer than 3% of the youths were interviewed in a secure program setting, such as residential commitment programs, county jails, or a juvenile detention center.

### 2.4 Measures

#### 2.4.1 Attitudes toward School Outcome

We used the Behavioral Assessment System for Children (BASC) (Reynolds & Kamphaus, 2005) attitudes to school questions in this study. This 12 item scale has been found to have very good psychometric properties (alpha = 0.83, retest reliability = 0.80). Table 1 presents the youths’ replies to each of the 12 questions about attitudes to school across the five time points of the study. As can be seen, at baseline there are a mixture of positive and negative attitudes towards school, although negative attitudes are more often reflected in the youths’ replies to these questions. For example, 80% of the truant youth report finishing school is important, and 53% indicate school feels good. On the other hand, 83% of the youth can’t wait until school is over, 56% don’t like thinking about school, 81% indicate they get bored in school, and 71% feel school has too many rules. There is a modest trend for attitudes favorable towards school to increase, and negative attitudes towards school to decrease over time.

Initially, when we were examining these attitudinal questions, we sought to conduct exploratory and confirmatory factor analyses of the 12 attitude measures. Then, growth modeling of the latent factors for attitudes about school would have been estimated. Due to the skewed question response patterns, relatively sizable number of items, and relatively small number of cases, however, factor analyses were inappropriate. Hence, the 12 attitude toward school questions were recoded such that a response reflecting a positive attitude towards school was coded as 1 and a negative attitude towards school was coded as 0. Then an additive index of the binary coded 12 school attitude items was created. As shown in Table 1, the mean scores for the attitudes toward school index increased across the time waves. The mean correlation among the five summed measures was *r =* .590, range *r* = .448 to .693.

#### 2.4.2 School Problem Behavior Data^1^

As part of our research collaboration, the Hillsborough County School District provided us with a variety of school information on youth in our project. These data files included school problem behavior data. We coded these data to correspond to the following time periods: (a) the year before entry into the project (baseline), 3-month follow-up (measured as the 90 days following last project participation [i.e., intervention session]), 6-month follow-up, 12-month follow-up, and 18-month follow-up. Since relatively few project youth attended summer school (<10%), this school session period was not included in our analyses.

Table 2 shows the percent of youth with recorded school behavior problems across the five time points of the project. A wide range of problem behaviors were recorded in the school records, ranging from problem behavior on a school bus to battery/fighting. Given the frequency distributions of the various reported behavior problems at the five time points, and the number of valid cases at the various time points, three problem behaviors had sufficient data to conduct meaningful analyses: (1) disobedience/insubordination, disrespectful, (2) inappropriate behavior, and (3) skipping class/left class without permission/left campus without permission. (For example, for disruptive behavior, nearly 90% of the youth had no record of this behavior problem at 3-month follow-up, 6-month follow-up and at 18-month follow-up; as well, 12% of 82 cases at 18-month follow-up yields only 10 cases engaging in this behavior for this time period.)

Depending on the distributions of the disobedience, inappropriate behavior and skipping variables at each time of the five time points, and where indicated, their initial skewness and kurtosis values, were either (1) treated as continuous variables without log transformation (prior year disobedience, prior year skipping), (2) log transformed to the base 10 to reduce skewness and kurtosis (with no record of having engaged in this behavior at a given time point scored as-1 [see discussion of the development of the self-reported delinquency measure in section 2.4.8]) (previous year inappropriate behavior, 3-month follow-up disobedience, 6-month follow-up disobedience, 12-month follow-up disobedience, 6-month follow-up inappropriate behavior, 12-month follow-up inappropriate behavior,18-month follow-up inappropriate behavior, and 12-month follow-up skipping ), or (3) treated as an ordinal variable (i.e., less than five categories) (3-month follow-up inappropriate behavior, 3-month follow-up skipping, 6-month follow-up skipping, 18-month follow-up skipping, and 18-month follow-up disobedience ).

#### 2.4.3 Background Covariates

A number of sociodemographic covariates were used in our study: (1) age (in number of years); (2) gender (0 = male, 1 = female); (3) race (1 = African American, 0 = other); and (4) ethnicity (1 = Hispanic, 0 = other). As mentioned above, the sample included youths and their families residing in the state of Florida. It is important to examine the influence of Hispanic ethnicity when dealing with such a sample. Therefore, covariates for Hispanic ethnicity, as well as African-American race, were included in the covariates. Table 3 shows the distributions of the youths’ demographic characteristics (age, gender, and ethnicity). Most of the youth were male; they averaged 14.8 years in age at entry into the study; and they were ethnically diverse.

#### 2.4.4 Who Youth Was Living With Covariate

Information was obtained from the youth and parent/guardian interviews regarding who the youth was living with. Relatively few youths were living with both of their biological parents (17%), birth father (3%), or grandparents (4%). On the other hand, a third of the youth were living with their mother alone. We created a variable reflecting youth residence with mother (1 = living with mother alone) or in another living arrangement (0 = other living arrangement) for our analysis.

#### 2.4.5 Family Income Covariate

At their baseline interviews, parents/guardians were asked for information regarding their annual family income. Overall, the families in the project had modest annual incomes. Nearly 40% of the families had an annual income of $25,000 or less, 23% reported annual incomes between $40,000 and $75,000, and 10% indicated their annual incomes were greater than $75,000. The median annual family income was between $25,001 and $40,000. A measure of family income was created where 1 = less than $5,000, 2 = $5,001-10,000, 3 = $10,001-25,000, 4 = $25001-40,000, 5 = $40,001-75,000, and 6 = more than $75,000.

#### 2.4.6 Family Experience of Stressful/Traumatic Events Covariate

The youths’ parents/guardians were asked at baseline interview to indicate if the youth or their family ever experienced various stressful or traumatic events. Large percentages of families reported one or more of these experiences, with unemployment of parent (50%), divorce of parents (39%), death of a loved one (58%), serious illness (31%), and a legal problem resulting in jail or detention (26%) being noteworthy. In addition, 49% of the parents/guardians reported other stressful/traumatic experiences (e.g., youth being placed in foster care, not having a relationship with father, fighting with brothers and sisters, losing the opportunity to obtain a driver's license, separation from mother). A measure of trauma was created by calculating the total number of traumatic events that each youth's parent/guardian reported. Overall, an average of 2.99 stressful/traumatic events were reported (SD = 1.76).

#### 2.4.7 Marijuana Use at Baseline Covariate

The main data collection instruments were the Adolescent Diagnostic Interview ([ADI], Winters & Henly, 1993), and the Adolescent Diagnostic Interview- Parent/Guardian ([ADI-P], Winters & Stinchfield, 2003). Both the ADI and ADI-P were designed to be delivered within a highly structured and standardized format (e.g., most questions are yes/no) to capture *DSM-IV* criteria for substance use disorders and related areas of functioning. The instruments consist of multiple questions for each abuse and dependence criterion, including frequency of drug use behaviors. Item construction was informed by the literature on structured interviews, advice from an expert panel, and feedback from field testers (Winters & Henly, 1993). *DSM* guidelines and results from the statistical analysis provided the basis for scoring rules. Reliability and validity studies, involving over 1000 drug clinic adolescents for the ADI and about 200 parents/guardians for the ADI-P, provide a wide range of psychometric evidence pertaining to inter-rater agreement, test-retest reliability, convergent validity (with clinical diagnoses), self-report measures, and treatment referral recommendations (Winters & Henly, 1993; Winters & Stinchfield, 2003).

Marijuana use was measured through self-report questions on the ADI and the results of urine tests (UA), which were administered at baseline interview. Urine specimens using the Onsite CupKit® urine screen procedure were also collected to assess recent drug use. For marijuana (THC), the positive threshold level is 50 ng/ml of urine, and the surveillance window is 3-4 days for infrequent users, 10 days for heavy users, and 30 days for chronic users and/or users with high body fat. The ADI questions probed the use of marijuana as: never, less than five times, or five or more times. The youth interviews included a question on the recency of marijuana use: past day, past 2 days, past week, past 2 weeks, past month, past 6 months, and more than 6 months ago. Given the 30 day surveillance window for detecting marijuana use via urine testing, we examined, at each time point, the concordance between the urine test results and youth reported recency of use of the drug within the past month. Results indicated the vast majority of youth at each time point reported recency of marijuana use within the surveillance window period. For the baseline marijuana use measure, 64% of youth claimed use of marijuana in the past 30 days.

We combined the self-reported marijuana use and marijuana urine test data into an overall measure of marijuana use involving six categories: (1) marijuana use denied and UA test for marijuana negative (7%); (2) marijuana use denied and UA test data missing (due to reasons beyond the youth's control [e.g., incarcerated]) (0.3%); (3) marijuana use denied and UA test data missing (not due to reasons beyond the youth's control [e.g., participant refusal]) (0.3%); (4) UA test missing or negative for marijuana, but youth reported marijuana use one to four times (17%); (5) UA test missing or negative, but youth reported marijuana use five or more times (29%); (6) UA test positive for marijuana (46%). Since there were relatively few cases in categories (2) and (3), they were combined with category (1) for further analysis, resulting in four categories of self-reported and UA marijuana use.

#### 2.4.8 Self-Reported Delinquent Behavior at Baseline Covariate

Based on the work of Elliott, Ageton, Huizinga, Knowles, and Canter (1983), we measured the youths' delinquent behavior prior to their interviews by asking how many times they engaged in each of 23 delinquent behaviors. Similar to Elliott et al. (1983), we developed five summary indices of delinquent involvement: general theft (e.g., petit theft, vehicle theft/joyriding, burglary); crimes against persons (e.g., aggravated assault, fighting, and robbery); index crimes (similar to UCR Index Part I); drug sales; and total delinquency (i.e., the sum of the 23 delinquent activities). Among the 300 youth, relatively high rates of delinquency were reported during the year prior to their initial interviews. High prevalence rates were reported for index offenses (50%), crimes against persons (75%), general theft (75%), drug sales (29%), and total delinquency (94%). Further, from 1% to 15% of the youths reported engaging in the offenses (represented by the various indices) 100 times or more; some reported many hundreds of offenses.

The range of responses to the items comprising the five self-reported delinquency scales was large, ranging from no activity to hundreds (and in few cases thousands), and analysis of the frequency data as an interval scale was not appropriate as a measure of involvement in delinquency/crime. Raw numbers of offenses do form an interval scale, which might be useful if one were predicting crime rates for populations. However, the difference between no offense and 1 offense is not the same as the difference between 99 and 100 offenses in terms of involvement. A transformation was employed so that equal intervals on the transformed scale would represent differences in involvement. We interpreted the differences between 1 and 10, 10 and 100, and 100 and 1,000 offenses as being comparable. Accordingly, we log transformed the number of offenses for each scale to the base 10.

For any base, logarithms exist for all positive numbers. The choice of base does not matter if the logarithms are analyzed by a statistical procedure invariant under linear transformation, such as analysis of variance, multiple regression, discriminant analysis, or factor analysis. However, regardless of the base, the logarithm of 0 does not exist. Some other method must be employed to determine the score assigned to no offenses. For any base, 0 is the logarithm of the value 1, and 1 is the logarithm of the base. If the difference from “base” offenses (10 in this study) to 1 offense is assigned the difference in logarithm scores of 1 and 0, this provides a unit of measurement for assigning a score even lower than 0—a negative number—to no offenses. In this study, a score of -1 was assigned. This evaluates the difference between no offense and 1 offense as equal in importance as the difference between 1 offense and 10, or between 10 offenses and 100 (Dembo & Schmeidler, 2002).

The correlation between the log transformed measure of total delinquency and the other delinquency measures was sizable and statistically significant at baseline (mean correlation = .603). Importantly, the skewness and kurtosis of the log transformed measure of total delinquency was dramatically lower than those of the untransformed measure. Hence, we decided to use the log transformed measure of total delinquency in our analyses.

#### 2.4.9 Sexual Risk Behavior at Baseline Covariate

We probed youths’ involvement in sexual risk behavior at baseline and at each follow-up interview using the POSIT HIV/STD Risk Behavior instrument. The POSIT 11-item HIV/STD risk scale was developed by the NOVA Research Company (Young & Rahdert, 2000). The instrument has been pilot tested and found to have very good psychometric properties (e.g., internal consistency = 0.80, one-week test-retest reliability = 0.90; concurrent validity with the Sexual Risk Questionnaire scores: r = 0.80). In the current study, the internal consistency (Cronbach's Alpha) of the 11 items was 0.73.

Table 4 presents the baseline (lifetime) data for the 11 sexual risk items. As can be seen, large percentages of youths reported close friends having had sex (78%), and 67% of the youths reported they had sexual intercourse. Importantly, sizable percentages of youths indicated they had sexual intercourse without using a condom (33%), and having 2 or more sexual partners (30%). In addition, 3% of the youths reported they ever had a sexually transmitted disease. Comparison of these results with findings reported in the Centers of Disease Control, 2009 Youth Risk Behavior Surveillance ([YRBS] CDC, 2011 http://www.cdc.gov/yrbs), indicates a much higher rate of ever having had sexual intercourse among youths in our study, than that reported by youths in the YRBS nationally (47%) or in Florida (48%--9^th^ grade = 31%; 10^th^ grade = 45%; 11^th^ grade = 57%). This result is consistent with the expectation that truant youth engage in sexual risk behavior at a higher rate than the general youth population.

Lack of condom use and number of sexual partners are also widely used sexual risk behavior measures in related research (Brook, Balka, Abernathy, & Hamburg, 1994; Bryan , Ray, & Cooper, 2007; Cooper, 2002; Elkington, Bauermeister, Brackis-Cott, Dolezal, & Mellins, 2009; Goldstein, Barnett, Pedlow, & Murphy, 2007; Komro, Tobler, Maldonado-Molina, & Perry, 2010; Morris, Baker, Valentine, & Pennisi, 1998; Morris, Harrison, Knox, Tromanhauser, & Marquis, 1995; Murphy, Brecht, Herbeck, & Huang, 2009; Wetherill & Fromme, 2007; also see: Warren et al., 1998; de Guzman & Bosch, 2007). Hence, we developed a summary measure involving the following four indicators reflecting the youths’ involvement in sexual risk behaviors at baseline: (1) had sexual intercourse, (2) had sexual intercourse without using a condom, (3) had sex with two or more people, and (4) had a sexually transmitted disease. Table 4 also shows the results for the summary index we developed from the youths’ replies to these questions (specific items in bold in table).

#### 2.4.10 Treatment Effects Covariates

Measures for treatment effects of the drug use intervention were also included in analyses. The overall Brief Intervention treatment effect was measured by a variable that contrasts youth and families receiving BI services (BI-Y + BI-YP) versus those receiving standard services (STS). In addition, specific comparisons were made between service conditions: (1) BI-Y vs. STS, (2) BI-YP vs. STS, and (3) BI-Y vs. BI-YP.

## 3. Analysis Strategy and Results

### 3.1 Strategy of Analysis for Attitude toward School Index

Latent growth modeling (see Duncan, Duncan, Strycker, Li, & Alpert, 1999) was used to examine growth in positive attitudes towards school over the five time points across 18 months. In latent growth models, longitudinal data are modeled as resulting from latent variables of a mean trend for the population (referred to as slope or trend) while allowing for differences among individuals (referred to as level or intercept). The growth model analyses were completed using Mplus Version 7.11 (Muthèn & Muthèn, 1998-2012), a versatile, multivariate statistical modeling program that estimates a variety of models for continuous and categorical observed and latent variables. Maximum likelihood estimation was used with these continuous data.

A non-significant chi-square value indicates an acceptable model fit. Mplus also provides a number of measures that aid in assessing the closeness of fit of the model to the data. Four fit indices were used to evaluate the model fit: (1) the comparative fit index (CFI) (Bentler, 1990); (2) the Tucker-Lewis coefficient (TLI) (Tucker & Lewis, 1973); (3) root mean square error of approximation (RMSEA) (Byrne, 2001); and (4) standardized root mean square residual (SRMR). The typical range for both TLI and CFI is between 0 and 1 (although TLI can exceed 1.0), with values greater than .95 indicating a good fit (Browne & Cudeck, 1993; Hu & Bentler, 1999). For RMSEA, values at .05 or less indicate a close model fit, and values between .05 and .08 suggest a mediocre model fit (Browne & Cudeck, 1993). For SRMR, values close to zero indicate a close model fit for both continuous and categorical outcomes (Yu & Muthèn, 2001). (For analyses involving weighted least squares estimation, weighted root mean square residual [WRMR] values close to .90 indicate a close fit of the model to the data [Schreiber, Stage, King, Nora, & Barlow, 2006]).

Because the time of entry into the study determined the number of follow-up interviews each youth and parent/guardian received, the data that are missing are a consequence of the study design. Accordingly, the Mplus feature allowing for maximum likelihood estimation of missing values was used to treat the missing data (Muthèn & Muthèn, 1998-2012).

### 3.2 Attitude toward School Growth Model

**Figure 1:**
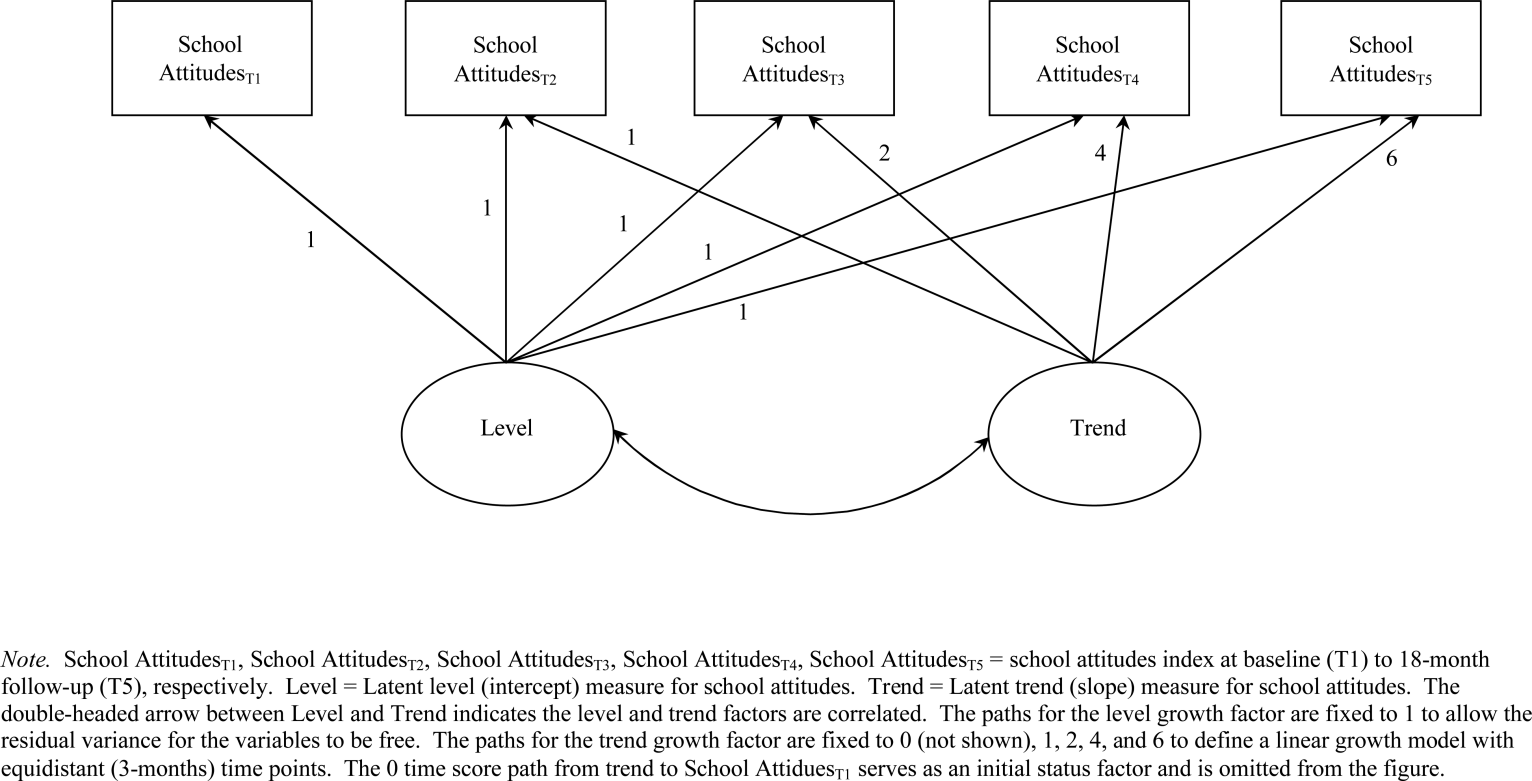
Linear Growth Model for Positive Attitude toward School *Note.* School Attitudes_T1_, School Attitudes_T2_, School Attitudes_T3_, School Attitudes_T4_, School Attitudes_T5_ = school attitudes index at baseline (T1) to 18-month follow-up (T5), respectively. Level = Latent level (intercept) measure for school attitudes. Trend = Latent trend (slope) measure for school attitudes. The double-headed arrow between Level and Trend indicates the level and trend factors are correlated. The paths for the level growth factor are fixed to 1 to allow the residual variance for the variables to be free. The paths for the trend growth factor are fixed to 0 (not shown), 1, 2, 4, and 6 to define a linear growth model with equidistant (3-months) time points. The 0 time score path from trend to School Attidues_T1_ serves as an initial status factor and is omitted from the figure.

Linear and quadratic growth models were fit to the positive attitudes toward school index, BASC, longitudinal data. Although both quadratic and linear growth models were consistent with the data, the linear model solution had a closer fit, reflected in a lower Bayesian Information Criterion (BIC) value, than the quadratic growth model (5392.16 versus 5407.26), respectively. Other model fit measures (i.e., RMSEA, CFI/TLI, and SRMR) were similar in value. In the interest of parsimony, the linear growth model was used for further analyses. Figure 1 presents the linear growth model we estimated.

Table 5, which presents these results, indicates a positive trend in attitudes toward school over time. There was a negative covariance between trend and level, indicating an overall decline in positive attitudes towards school over time, given one's baseline attitudes towards school.

### 3.3 Sociodemographic and Intervention Condition Predictors of Growth Model Latent Variables

We completed an overall, covariate analysis of the data. The covariate analysis examined covariate effects on the growth model for school attitudes. The covariates included age, gender, family income level, living with mother, being African-American, Hispanic, family experience of stressful/traumatic events, sexual risk behavior at baseline, self-reported delinquency at baseline, marijuana use at baseline, and an overall intervention effect involving four models: Model 1 = BI (BI-Y + BI-YP) vs. STS; Model 2 = BI-Y vs. STS; Model 3 = BI-YP vs. STS; and Model 4 = BI-YP vs. BI-Y.

As the results in Table 6 show, several consistent level and trend covariate effects were found across the four model comparisons. In regard to level, there were significant, positive relationships between age and positive attitudes toward school for Models 1, 3 and 4. There was a significant, positive relationship between being African-American and attitudes toward school at baseline (level) for Models 1-4. In addition, there was a significant, negative relationship for level between self-reported delinquency at baseline and positive attitudes toward school in each of the four models. One significant trend effect was found. For Models 1, 2 and 4, African-American youth experienced a lower rate of increase in positive attitudes toward school over time, than non-African-American (mainly White) youth.

According to these findings, older youth and African-American youth were significantly more likely to report more positive attitudes toward school at baseline. In contrast, youth reporting more involvement in delinquency reported significantly less positive attitudes toward school at baseline, than youth reporting less involvement in delinquent behavior. In addition, as noted above, the rate of change in positive attitudes toward school was significantly lower among African-American youth, compared to other youth in the study. Importantly, no significant Brief Intervention level or trend effects were found in any of the comparisons shown in Table 6.

There was a possible assessment validity problem for 13 follow-up interview cases completed by a former staff member. These assessments occurred during the first two years of the project and involved several assessments at each follow-up. The analyses reported in the present paper were re-computed with the thirteen cases suspected of having validity issues excluded. The results were unaffected by excluding these cases. The results reported include these 13 cases in the analyses.

### 3.4 Strategy of Analysis for School Misbehavior Measures

**Figure 2:**
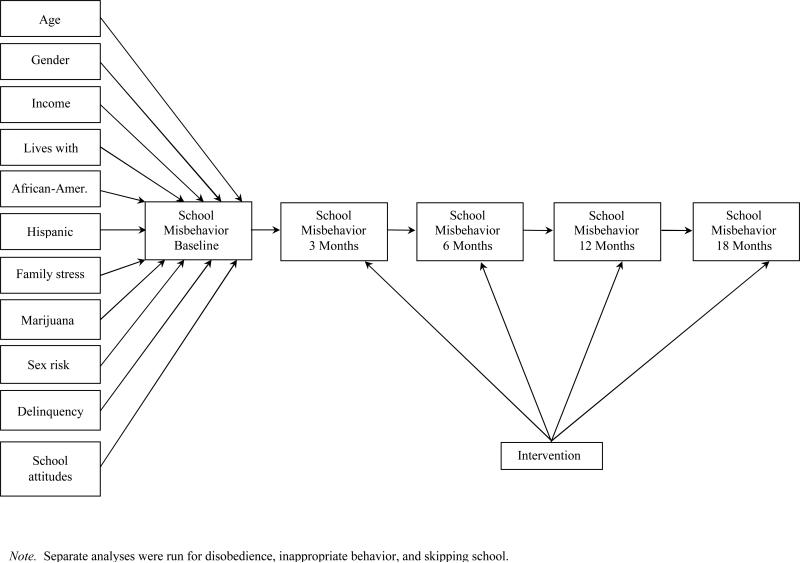
The Basic Auto-Regressive Lag Model for School Behavior Problems *Note.* Separate analyses were run for disobedience, inappropriate behavior, and skipping school.

Since the items for school misbehavior for disobedience, inappropriate behavior, and skipping school involved both continuous and categorical variables, and, hence, were not measured on the same scale, it was not possible to estimate growth models for these data. We decided to conduct regression analyses to examine the relative predictive ability of the variables discussed in the methods section on school disobedience, inappropriate behavior, and skipping school across the five time points. The regression analyses were run using Mplus version 7.11 (Muthén & Muthén, 1998-2012). The inappropriate behavior and skipping school regression analyses involved WLSMV estimation (a robust weighted least squares estimator using a diagonal weight matrix). The disobedience analyses involved MLR estimation (maximum likelihood parameter estimates with standard errors and a chi-square statistic [when applicable]) that are robust to non-normality. The MLR standard errors are computed using a sandwich estimator. The MLR chi-square test statistic is asymptotically equivalent to the Yuan-Bentler T2* test statistic (p. 533). Because the time of entry into the study determined the number of follow-up school misbehavior data points we used, the data that are missing are a consequence of the study design. Accordingly, the Mplus feature allowing for maximum likelihood estimation of missing values was used to treat the missing data (Muthèn & Muthèn, 1998-2012). Given the time ordering of the school misbehavior variables, an auto-regressive lag model was estimated. As a preliminary analysis step, we first estimated, for each school misbehavior, the basic model depicted in Figure 2.

As Figure 2 shows, the covariates of age, gender, family income level, who youth lives with, being African-American, Hispanic, family stress/trauma experiences, marijuana use at baseline, sexual risk behavior at baseline, self-reported total delinquency at baseline, and the summary measure of attitudes toward school at baseline were specified to influence, separately, the baseline school misbehaviors. For each of the school misbehaviors (disobedience, inappropriate behavior, skipping school), each of the follow-up misbehavior time points was regressed on its preceding time point school misbehavior. Finally, an overall STS vs. BI [BI-Y+BI-YP] intervention effect was specified on school misbehavior at 3-month, 6-month, 12-month, and 18-month follow-up.

Three basic model estimates were completed, one for each of the school misbehaviors. Results indicated an acceptable fit of the model to the data for each misbehavior: (1) disobedience (chi-square = 62.92, df = 49, *p* = 0.09; RMSEA = 0.031; SRMR = 0.063); (2) inappropriate behavior (chi-square = 53.08, df = 51, *p* = 0.39; RMSEA = 0.012; WRMR = 0.780; (3) skipping school (chi-square = 46.88, df = 51, *p* = 0.64; RMSEA = 0.000; WRMR = 0.737). Similar to the attitude toward school analyses, we estimated four autoregressive lag models for each school misbehavior: Model 1 = an overall intervention effect (BI-Y + BI-YP = 1, STS = 0); Model 2 = comparison of the BI-Y vs. STS conditions; Model 3 = comparison of the BI-YP vs. STS conditions, and Model 4 = comparison of the BI-YP vs. BI-Y conditions. For these analyses, the directional hypotheses were considered significant at the .05 level by a one-tailed test.

Since the analysis of each model for each outcome included tests of four follow-up time periods, the Holm procedure was used to adjust significance for multiple tests of significance (Holm, 1979). This refinement of the Bonferroni procedure requires the same significance level for the most significant test (for these analyses with four tests, .05 / 4 = .0125), but larger, less restrictive, significance levels for the others. To facilitate application of this procedure, in Tables 7-9, one-sided significance of levels *p* < .05 and *p* < .0125 are indicated for intervention effects.

The modification indices for one or more of the model comparisons for each school misbehavior indicated the model fit could be improved by including additional covariates at one or more time points in the model. These additions are indicated in the respective tables reporting the results of these analyses.

### 3.5 Intervention Group Comparison of Demographic and Other Covariates

We compared the STS, BI-Y, and BI-YP groups in regard to their baseline background and other covariates used in the attitudes to school analyses. No significant differences were found for each of these variables. (These results have been omitted due to space concerns. A copy is available from the lead author upon request.)

### 3.6 Covariate Predictors of School Misbehaviors: Disobedience

#### 3.6.1 Assessment of Model 1 (BI-Y + BI-YP = 1 vs. STS = 0)

Table 7 displays the results of our estimation of Model 1. The model fit to the data was reported above (chi-square = 62.92, df = 49, *p* = 0.09; RMSEA = 0.031; SRMR = 0.063). Findings indicated significant positive effects for age (being younger), African-American, Hispanic, and self-reported involvement in delinquency on baseline (past year) disobedient behavior. Baseline disobedient behavior was significantly and positively related to disobedient behavior at 3-month follow-up. Age (being younger) and disobedient behavior (positive relationship) at 3-month follow-up were significantly related to disobedient behavior at 6-month follow-up. Disobedient behavior at 6-month follow-up was significantly and positively related to disobedient behavior at 12-month follow-up. In addition, age (being younger) was significantly related to being involved in disobedient behavior at 18-month follow-up.

No significant intervention effect was found. Contrary to the hypothesis, youth receiving BI services (BI-Y + BI-YP) were more likely to be involved in disobedient behavior at 6-month follow-up, than youth receiving STS (estimate = .126, S.E. = 0.074, *p* = .957 by the one-sided test). If a two-sided test had been performed, this result would not have been significant (*p* = .086).

#### 3.6.2 Assessment of Model 2 (BI-Y = 1 vs. STS = 0)

Table 7 also displays the results of our estimation Model 2. The model fit to the data was quite good (chi-square = 57.74, df = 47, *p* = 0.14, RMSEA = 0.034; SRMR = 0.068). Findings indicated significant positive effects on African-American, Hispanic, and self-reported delinquency on baseline disobedient behavior. There were also significant autoregressive effects on disobedient behavior at successive time points (baseline disobedience on 3-month follow-up disobedience; 3-month follow-up disobedience on 6-month follow-up disobedience; 6-month follow-up disobedience on 12-month follow-up disobedience). Age (being younger) was significantly related to disobedience at 6-month follow-up and 18-month follow-up. Family experience of stressful/traumatic events was significantly and negatively related to disobedience at 6-month follow-up. There was no significant intervention effect on disobedience at any follow-up time point.

#### 3.6.3 Assessment of Model 3 (BI-YP = 1 vs. STS = 0)

The results of our estimation of Model 3, comparing BI-YP and STS youth, are also shown in Table 7. The model fit to the data was good (chi-square = 63.36, df = 47, *p* = 0.06, RMSEA = 0.000; SRMR = 0.085). Findings indicated a significant positive effect of family income, being African-American, and Hispanic on baseline disobedient behavior. Significant autoregressive effects were found for baseline disobedient behavior on 3-month follow-up disobedient behavior and 3-month follow-up disobedient behavior on 6-month follow-up disobedient behavior. Age (being younger) was significantly related to disobedience at 18-month follow-up. Youth living with mother only were significantly more likely to be involved in disobedient behavior at 3-month follow-up.

There was a significant intervention effect on disobedience at 3-month follow-up. Youth receiving BI-YP services were less likely to be cited for disobedience, than youth receiving STS (estimate = -0.145, S.E. = 0.087, *p* = .048; effect size = -0.30, small) (Muthèn & Asparouhov, 2002). Note that this result was not significant by the Holm procedure, which requires *p* < .0125. Contrary to the hypothesis, at 6-month follow-up, youth receiving BI-YP services were more likely to be cited for disobedience, than youth receiving STS (estimate = 0.145, S.E. = 0.083, *p* = .040 by the one-sided test). If a two-sided test had been performed, this result would not have been significant (*p* = .080).

#### 3.6.4 Assessment of Model 4 (BI-YP = 1 vs. BI-Y = 0)

The results of our estimation of Model 4, comparing BI-YP and BI-Y youth, indicated a good fit of the model to the data (chi-square = 66.90, df = 51, *p* = 0.07, RMSEA = 0.040; SRMR = 0.073). The Model 4 results, shown in Table 7, comparing youth in the BI-Y vs. BI-YP conditions, indicated being African-American is significantly related to baseline disobedience. Two significant autoregressive effects were found: 3-month follow-up disobedience on baseline disobedience and 6-month disobedience on 3-month disobedience.

A significant intervention effect was found. Youth receiving BI-YP services were less likely to be cited for disobedience at 3-month follow-up, than youth receiving BI-Y services (estimate = -0.143, S.E. = 0.085, *p* = .047; effect size = -0.30, small) (Muthèn & Asparouhov, 2002). Note that this result was not significant by the Holm procedure, which requires *p* < .0125.

### 3.7 Covariate Predictors of School Misbehaviors: Inappropriate Behavior

#### 3.7.1 Assessment of Model 1 (BI-Y + BI-YP = 1 vs. STS = 0)

Table 8 displays the results of our estimation of Model 1. The model fit to the data was good (chi-square = 53.08, df = 51, *p* = 0.39; RMSEA = 0.012; WRMR = 0.780). Findings indicated significant effects for age (being younger), gender (being male), being African-American, and reported involvement in sexual risk behavior at baseline and baseline (past year) inappropriate behavior. Significant autoregressive effects for inappropriate behavior were found between inappropriate behaviors at baseline and 3-month follow-up, 3-month follow-up and 6-month follow-up, 6-month follow-up and 12-month follow-up, and 12-month follow-up and 18-month follow-up.

A significant intervention effect was also found. Youth receiving BI services (BI-Y + BI-YP) were less likely to be involved in inappropriate behavior at 12-month follow-up, than youth receiving STS (estimate = -0.208, S.E. =0.125, *p* = .047; estimated effect size = -0.50, moderate) (Muthèn & Asparouhov, 2002). Note that this result was not significant by the Holm procedure, which requires *p* < .0125.

#### 3.7.2 Assessment of Model 2 (BI-Y = 1 vs. STS = 0)

Table 8 also displays the results of our estimation of Model 2. The model fit to the data was quite good (chi-square = 49.84, df = 51, *p* = 0.52, RMSEA = 0.000; WRMR = 0.753). Findings indicated significant effects for age (being younger), gender (being male), involvement in sexual risk behavior at baseline, and having negative attitudes toward school at baseline and baseline inappropriate behavior. There were also significant autoregressive effects on inappropriate behavior at each successive time point (baseline inappropriate behavior on 3-month follow-up inappropriate behavior; 3-month follow-up inappropriate behavior on 6-month follow-up inappropriate behavior; 6-month follow-up inappropriate behavior on 12-month follow-up inappropriate behavior; and 12-month follow-up inappropriate behavior on 18-month follow-up inappropriate behavior). There were no significant intervention effects on inappropriate behavior at any of the follow-up time points.

#### 3.7.3 Assessment of Model 3 (BI-YP = 1 vs. STS = 0)

The results of our estimation of Model 3, comparing BI-YP and STS youth, are also shown in Table 8. The model fit to the data was good (chi-square = 48.96, df = 51, *p* = 0.55, RMSEA = 0.000; WRMR = 0.750). Findings indicated significant effects of age (being younger), gender (being male), being African-American, involvement in sexual risk behavior at baseline, and self-reported involvement in delinquency at baseline on baseline inappropriate behavior. Significant autoregressive effects were found for baseline inappropriate behavior on 3-month follow-up inappropriate behavior, 3-month follow-up inappropriate behavior on 6-month follow-up inappropriate behavior, 6-month follow-up inappropriate behavior on 12-month follow-up inappropriate behavior, and a marginally significant effect (*p* = .054) of 12-month follow-up inappropriate behavior on 18-month follow-up inappropriate behavior.

No significant intervention effects were found. However, there was a marginally significant effect (*p* = .074) between BI-YP and inappropriate behavior at 18-month follow-up. Youth receiving BI-YP services were less likely to be cited for inappropriate behavior at 12-month follow-up, than STS youth.

#### 3.7.4 Assessment of Model 4 (BI-YP = 1 vs. BI-Y = 0)

The results of our estimation of Model 4, comparing BI-YP and BI-Y youth, indicated a good fit of the model to the data (chi-square = 47.27, df = 51, *p* = 0.62, RMSEA = 0.000; WRMR = 0.725). The Model 4 results shown in Table 8, comparing youth in the BI-Y vs. BI-YP conditions, indicated age (being younger), gender (being male), and involvement in sexual risk behavior at baseline are significantly related to baseline inappropriate behavior. Significant autoregressive effects were found for baseline inappropriate behavior on 3-month follow-up inappropriate behavior, 3-month follow-up inappropriate behavior on 6-month follow-up inappropriate behavior, and 6-month follow-up inappropriate behavior on 12-month follow-up inappropriate behavior.

A significant intervention effect was also found. Youth receiving BI-YP services were less likely to be cited for inappropriate behavior at 6-month follow-up, than youth receiving BI-Y services (estimate = -0.156, S.E. = 0.093, *p* = .046; effect size = 0.43, moderate) (Muthèn & Asparouhov, 2002). Note that this result was not significant by the Holm procedure, which requires *p* < .0125.

### 3.8 Covariate Predictors of School Misbehaviors: Skipping School

#### 3.8.1 Assessment of Model 1 (BI-Y + BI-YP = 1 vs. STS = 0)

Table 9 displays the results of our estimation of Model 1. The model fit to the data was very good (chi-square = 46.88, df = 51, *p* = 0.64; RMSEA = 0.000; WRMR = 0.737). Findings indicated significant effects for gender (being male) and involvement in sexual risk behavior at baseline on baseline (past year) skipping of school. In addition, significant autoregressive effects were found for baseline skipping behavior on 3-month follow-up skipping behavior, 3-month follow-up skipping behavior on 6-month follow-up skipping behavior, and 6-month follow-up skipping behavior on 12-month follow-up skipping behavior. No significant intervention effects were found.

#### 3.8.2 Assessment of Model 2 (BI-Y = 1 vs. STS = 0)

Table 9 also displays the results of our estimation of Model 2. The model fit to the data was quite good (chi-square = 40.02, df = 51, *p* = 0.87, RMSEA = 0.000; WRMR = 0.641). Findings indicated a significant positive effect of gender (being male) and involvement in sexual risk behavior at baseline on baseline skipping behavior. There were also significant autoregressive effects on disobedient behavior at successive time points (baseline skipping on 3-month follow-up skipping; 3-month follow-up disobedience on 6-month follow-up skipping; and 6-month follow-up skipping on 12-month follow-up skipping). There was no significant intervention effect on skipping at any follow-up time point.

#### 3.8.3 Assessment of Model 3 (BI-YP = 1 vs. STS = 0)

The results of our estimation of Model 3, comparing BI-YP and STS youth, are also shown in Table 9. The model fit to the data was good (chi-square = 40.63, df = 51, *p* = 0.85, RMSEA = 0.000; WRMR = 0.648). Findings indicated no significant covariate effects on baseline skipping behavior. On the other hand, significant autoregressive effects for skipping behavior were found for each successive time point (baseline on 3-month follow-up, 3-month follow-up on 6-month follow-up, 6-month follow-up on 12-month follow-up, and 12-month follow-up on 18-month follow-up). There were no significant intervention effects on skipping behavior at any follow-up time point.

#### 3.8.4 Assessment of Model 4 (BI-YP = 1 vs. BI-Y = 0)

The results of our estimation of Model 4, comparing BI-YP and BI-Y youth, indicated a good fit of the model to the data (chi-square = 43.51, df = 51, *p* = 0.76, RMSEA = 0.000; WRMR = 0.696). The Model 4 results shown in Table 9, comparing youth in the BI-Y vs. BI-YP conditions, indicated being Hispanic was significantly related to baseline skipping behavior. Three significant autoregressive effects for skipping behavior were found: baseline skipping on 3-month follow-up skipping, 3-month follow-up skipping on 6-month follow-up skipping, and 6-month follow-up skipping on 12-month follow-up skipping. No significant intervention effects were found.

### 3.9 Intervention Group Comparison of Demographic and Other Covariates

We compared the STS, BI-Y, and BI-YP groups in regard to their baseline background and other covariates used in the school behavior problem analyses. One significant effect was obtained for past year disobedience (F = 3.08, df = 2.296, *p* = .047). However, this effect was not significant by the Holm procedure, taking the number of covariates into account. (These results have been omitted due to space concerns. A copy is available from the lead author upon request).

## 4. Discussion

This study had two primary aims. First, it examined the continuity in truant youths' positive attitudes toward school and their engagement in misbehavior in school (disobedience, inappropriate behavior, and skipping behavior), separately, over five time points spanning 18 months. Growth model parameterization was used to examine changes in positive attitudes toward school, but it could not be used to examine school misbehavior. Instead, auto-regressive lag models were estimated for the school misbehavior outcomes. Second, this study sought to examine the influence of the BI on growth in attitudes toward school and misbehavior in school, separately, controlling for various correlates of truancy. In particular, it was hypothesized participants in the BI would have more positive attitudes toward school and less misbehaviors in school than those who received STS.

Four hypotheses were articulated regarding the second aim of this study to test the effects of the intervention on changes in school attitudes and misbehavior over time. First, youths receiving BI intervention services (either BI-Y or BI-YP) will show a greater increase in favorable attitudes toward school over time, than youth receiving STS services, controlling for socio-demographic and background covariates. Second, youths receiving BI intervention services (either BI-Y or BI-YP) will show a greater reduction in problem behavior in school, than youth receiving STS services, controlling for socio-demographic and background covariates. Third, youths receiving BI-YP intervention services will show a greater increase in favorable attitudes toward school over time, than youth receiving BI-Y services, controlling for socio-demographic and background covariates. Fourth, youths receiving BI-YP intervention services will show a greater reduction in problem behavior in school, than youth receiving BI-Y services, controlling for demographic and background covariates.

With respect to the first primary aim, a linear growth model was found to fit the attitudes toward school longitudinal data. There was evidence to suggest the youths’ attitudes toward school at various time points were linearly related across time. In regard to the truant youths’ attitudes toward school, we found, at baseline, a mixture of positive and negative attitudes towards school, with more negative, than positive attitudes, towards school being reflected in their answers to these questions. There was indication of an increase in positive attitudes toward school over time.

Further, we found longitudinal trends in school misbehaviors for the truant youth. As reported earlier, a wide range of problem behaviors were recorded in the school records, ranging from problem behavior on a school bus to battery/fighting. However, three problem behaviors had sufficient data across the five time points to conduct meaningful analyses: (1) disobedience/insubordination and disrespectful, (2) inappropriate behavior, and (3) skipping class/left class without permission/left campus without permission. Since the items for school misbehavior for disobedience, inappropriate behavior, and skipping involved both continuous and categorical variables, and, hence, were not measured on the same scale, growth model estimation was not possible for these data. Given the time ordering of the school misbehavior variables, auto-regressive lag models were estimated separately for the three types of misbehavior. Overall, significant, positive and consistent auto-regressive effects were found for each of the school misbehaviors, indicating that, once initiated, youth continue to engage in them.

With respect to the four hypotheses regarding the impact of the intervention on the growth or continuity in attitudes toward school and school misbehaviors, the hypotheses were generally unsupported. Several socio-demographic covariate effects on the youths’ attitudes towards school over time were found. At baseline, older youth held more favorable attitudes toward school, and youth involved in delinquency held less favorable attitudes toward school. African-American youth held more favorable attitudes toward school at baseline, but had a lower rate of increase in favorable attitudes toward school, than other youth in the study, over time. Importantly, however, no significant, overall BI effect was uncovered. Additional analyses, comparing three different intervention conditions (STS vs. BI-Y, STS vs. BI-YP, and BI-Y vs. BI-YP) also found no significant level or trend effects.

Although there were some statistically significant intervention effects at specific follow-ups, none of the three school misbehaviors were significant when applying the Holm procedure, taking account of the four follow-ups. Thus, our results provide no support for intervention effects on school behaviors. It is important to keep in mind that the BI program does not contain any specific content focus on school attitudes or behavior in school, so the absence of an intervention effect in these areas is not surprising.

Despite the lack of an intervention effect, this study makes an important contribution to the literature on truancy. Most studies of truant populations are cross-sectional, and simply examine the correlates of truancy. This study is one of very few that examines longitudinal data for truants. Indeed, surprisingly little is known about truants and their school-related behaviors and attitudes. As our analyses indicate, truants generally hold unfavorable attitudes towards school and are involved in a variety of problem behaviors at school over time. They represent a high risk group for school dropout and causing management and discipline problems to school officials. However, sanction-oriented approaches to address these issues, and zero tolerance policies, have been shown to be ineffective in reducing truancy (Caton, 2012; Center for Mental Health in Schools at UCLA, 2011; Noguera, 2009; Sprott, Jenkins & Doob, 2005). There is a serious need to provide effective intervention services to truant youth.

Quality assessments and involvement in effective intervention programs are needed. In this vein, intervention programs need to address the experience that truant youth are heterogeneous in regard to their problems and service needs. They present different risk profiles, reflecting various socio-demographic and psychosocial factors that need to be considered in efforts to remediate their problem behaviors (Maynard et al., 2012). As well, effective truancy reduction efforts require the support of multiple sources (schools, family, community and social services) (Edwards, 2005). It is hoped that our results will encourage developers of brief interventions to incorporate these features in their services.

There were a few limitations to this study. First, there were limitations due to the nature of the sample, which consisted, primarily, of truant youth picked up by law enforcement or placed in a diversion program. Hence, the results of the study may not generalize to truant youth who do not have such agency contact or involvement. Second, the truant youth were living in one geographical area. It would be helpful for researchers to consider future studies examining the effects of interventions focusing on one domain of at-risk behavior may have on other risk behavior among youth living in diverse socio-demographic circumstances.

## Figures and Tables

**Table 1:** Truant Youth Attitudes toward School Outcome over Time

BASC Items	Baseline (n=299 to 300)	3-Month Follow-up (n=267 to 269)	6-Month Follow-up (n=253 to 255)	12-Month Follow-up (n=201 to 203)	18-Month Follow-up (n=162 to 165)
1. Finishing my school work is important to me.	80%	89%	91%	91%	96%
2. I can hardly wait to quit school.	33%	29%	24%	22%	22%
3. I can't wait for school to be over.	83%	80%	78%	78%	72%
4. I don't care about school.	22%	13%	11%	12%	9%
5. I don't like thinking about school.	56%	52%	54%	56%	43%
6. I get bored in school.	81%	76%	75%	71%	69%
7. I hate school.	35%	26%	25%	20%	18%
8. I wish there were no report cards.	50%	45%	42%	36%	31%
9. My school feels good to me.	53%	60%	59%	62%	74%
10. School has too many rules.	71%	63%	64%	61%	61%
11. School is a waste of time.	15%	9%	9%	9%	7%
12. School is boring.	69%	66%	63%	63%	58%

Attitudes Toward School Index:					
Mean:	6.17	6.91	7.06	7.24	7.84
SD:	3.09	3.00	2.94	2.95	2.80

**Table 2:** Percent of Truant Youth with One or More School Recorded Problem Behaviors for 1 Year Prior to Project Entry and for Each Follow-up Period

Problem Behavior Variables	Prior Year (n=299)	3-Month Follow-up (n=201)	6-Month Follow-up (n=157)	12-Month Follow-up (n=122)	18-Month Follow-up (n=82)
Drug possession and/or use	6%	2%	2.5%	0.8%	2.4%
School bus-Inappropriate behavior	12%	2.5%	3.8%	4.1%	1.2%
School bus-Other problem behavior	7%	0.5%	2.5%	1.6%	3.7%
**Disobedience/insubordination, disrespectful**	51.2%	26.9%	25.5%	39.3%	29.3%
Disruptive (or continuous disruptive behavior/expulsion)	29.8%	10.4%	10.8%	17.2%	12.2%
**Inappropriate behavior**	50.8%	20.9%	19.7%	42.6%	20.7%
Battery/fighting	7.7%	2.5%	1.9%	4.9%	2.4%
Bullying/harassment, threat/intimidation	5.4%	1.0%	.6%	2.5%	2.4%
Non-compliance with assigned discipline	15.4%	5%	5.1%	6.6%	3.7%
Profanity	16.1%	8.5%	7.6%	12.3%	7.3%
**Skipping class/left class without permission/left campus without permission**	38.5%	17.4%	15.3%	22.1%	14.6%
Tardiness	33.8%	15.4%	10.8%	16.4%	14.6%
Other	19.7%	6.5%	7.6%	11.5%	9.8%
Truancy	0.3%	--	0.6%	--	--
Drug Sales	--	--	--	--	--

*Note*. “Other” includes school violations relating to: dress code, non-controlled substance, other minor incidents, falsification of records, petty theft, sexual offense, tobacco possession and/or use, disorderly conduct, larceny/theft, motor vehicle theft, weapons possession, vandalism. Bolded items were used in subsequent analyses.

**Table 3:** Background Characteristics of the Youths at Baseline (n = 300)

Covariates	
Age:	
Mean =	14.80
Standard Deviation =	1.30
Gender:	
Male	63.0%
Female	37.0%
	100.0%
Ethnicity/Race:	
Asian	1.0%
African American	25.7%
Hispanic	28.7%
Native American	0.3%
Caucasian	37.3%
Other (Mixed race)	7.0%
	100.0%
Who Youth Lives With:	
Both birth mother and birth father	16.7%
Birth mother alone	33.3%
Birth mother with stepfather/boyfriend	23.0%
Birth mother with relative or friend	10.3%
Birth father alone	2.7%
Birth father with stepmother or girlfriend	4.3%
Birth father with relative or friend	<1%
Adoptive parent(s)	2.7%
Grandparent(s)	4.3%
Other relative(s)	1.7%
Other	<1%
	100.0%
Family Annual Income Level (n = 297):	
Less than $5,000	5.1%
More than $5,000 up to $10,000	8.1%
More than $10,000 up to $25,000	26.3%
More than $25,000 up to $40,000	27.9%
More than $40,000 up to $75,000	22.9%
More than $75,000	9.8%
	100.0%
Family Experience of Stressful/Traumatic Events:	
Unemployment of parent	50.3%
Divorce of parents	38.7%
Death of loved one	57.7%
Serious illness	31.0%
Victim of violent crime	17.3%
Eviction from house or apartment	17.0%
Legal problem resulting in jail time or detention	26.4%
Accidental injury requiring hospitalization	12.0%
Other stressful/traumatic event	48.8%
Mean =	2.99
Standard Deviation =	1.76
Marijuana Use:	
Denied use and urine test results negative/missing	7.6%
Reported use 1-4 times and urine test negative/missing	17.0%
Reported use 5 or more times and urine test negative/missing	29.3%
Urine test positive	46.0%
	99.9%
Self-Reported Delinquency:	
Mean =	1.09
Standard Deviation =	0.87
Treatment Group:	
Brief Intervention-Youth (BI-Y)	33.7%
Brief Intervention-Youth plus Parent (BI-YP)	33.0%
Standard Truancy Services (STS)	33.3%
	100.0%

*Note*. Percentages may not add up to 100% due to rounding errors.

**Table 4:** Self-Reported Sexual Risk Behaviors at Baseline

Sexual Risk Behaviors	Ever (%) (n = 299)
1. Have any of your close friends had sex?	78
2. Have you had any kind of sexual contact with another person?	80
**3. Have you had sexual intercourse?**	67
**4. Have you had sexual intercourse without using a condom?**	33
5. Do you find it difficult to use condoms every time you have sex?	12
6. Have you thought you or your partner might be pregnant?	27
7. Have you been or gotten someone pregnant?	5
8. Have you been tested for HIV?	17
**9. Have you had sex with two or more people?**	30
10. Have you had anal intercourse (sex in the butt)?	5
**11. Have you had a sexually transmitted disease (STD)?**	3

Number of Sexual Risk Behaviors: Baseline	
0	32.4%
1	23.7%
2	23.7%
3	18.7%
4	1.3%

*Note*. Bolded items were used as indicators in the summary measure reflecting involvement in sexual risk behavior for subsequent analyses.

**Table 5:** Linear Growth Model of Youths’ Attitudes to School over Time, Unstandardized Estimates

Maximum Likelihood Estimates	Estimate	S.E.	Critical Ratio
Level:			
Attitudes to School T1	1.000	0.000	999.000
Attitudes to School T2	1.000	0.000	999.000
Attitudes to School T3	1.000	0.000	999.000
Attitudes to School T4	1.000	0.000	999.000
Attitudes to School T5	1.000	0.000	999.000
Trend:			
Attitudes to School T1	0.000	0.000	999.000
Attitudes to School T2	1.000	0.000	999.000
Attitudes to School T3	2.000	0.000	999.000
Attitudes to School T4	4.000	0.000	999.000
Attitudes to School T5	6.000	0.000	999.000
Covariances:			
Trend with Level	−0.376^**^	0.113	−3.314
Means:			
Level	6.398^***^	0.171	37.501
Trend	0.197^***^	0.034	5.859
Intercepts:			
Attitudes to School T1	0.000	0.000	999.000
Attitudes to School T2	0.000	0.000	999.000
Attitudes to School T3	0.000	0.000	999.000
Attitudes to School T4	0.000	0.000	999.000
Attitudes to School T5	0.000	0.000	999.000
Variances:			
Level	6.847^***^	0.714	9.586
Trend	0.088^**^	0.030	2.937
Residual Variances:			
Attitudes to School T1	3.296^***^	0.427	7.718
Attitudes to School T2	2.636^***^	0.332	7.933
Attitudes to School T3	2.892^***^	0.338	8.561
Attitudes to School T4	3.179^***^	0.422	7.536
Attitudes to School T5	3.108^***^	0.663	4.685
*R*^2^:			
Attitudes to School T1	0.675^***^	0.038	17.601
Attitudes to School T2	0.701^***^	0.035	20.160
Attitudes to School T3	0.663^***^	0.034	19.283
Attitudes to School T4	0.623^***^	0.044	14.220
Attitudes to School T5	0.639^***^	0.071	8.981

*Note*. Two-tailed *p*-values:

*p < .05

** *p* < .01

*** *p* < .001.

**Table 6:** Linear Growth Model Covariate Effects- Four Model Comparisons, Unstandardized Estimates

	Model 1 BI-Y+BI-YP vs. STS		Model 2 BI-Y vs. STS		Model 3 BI-YP vs. STS		Model 4 BI-YP vs. BI-Y	

*Covariates*	Level	Trend	Level	Trend	Level	Trend	Level	Trend
Age	0.389**	−0.006	0.235	−0.008	0.345*	0.010	0.583***	−0.010
Gender (1 = female)	0.101	0.017	0.304	−0.030	0.037	0.084	−0.140	0.003
Family income	−0.007	0.035	−0.080	0.023	0.122	0.025	−0.064	0.059
Youth lives with (1 = mother only)	0.158	−0.020	0.020	−0.034	−0.012	−0.015	0.379	0.003
Race (1 = African-American)	1.768***	−0.196*	1.507**	−0.216*	1.719**	−0.106	2.299***	−0.278**
Ethnicity (1 = Hispanic)	−0.201	0.151	−0.155	0.121	0.014	0.111	−0.351	0.206*
Family experience of stress/trauma	0.047	0.033	0.166	0.023	−0.045	0.046*	0.060	0.025
Sexual risk behavior at baseline	−0.254	0.040	−0.144	0.014	−0.214	0.045	−0.491**	0.057
Total delinquency at baseline	−0.754***	0.053	−0.908***	0.039	−0.757**	0.046	−0.656**	0.092
Marijuana use at baseline	0.071	−0.024	0.132	−0.034	0.088	−0.036	0.017	−0.016
Intervention (1 = Intervention)	−0.034	−0.019	−0.223	0.001	0.074	−0.029	0.296	−0.040

*Note*. For each model, the effects of the covariate for the Brief Intervention (BI) conditions on school attitudes vary, with Model 1 examining the effects of either BI (BI-Youth [BI-Y] + BI-Youth plus Parent [BI-YP]) vs. Standard Truancy Services (STS), Model 2 examining the effects of BI-Y vs. STS, Model 3 examining the effects of BI-YP vs. STS, and Model 4 examining the effects of BI-YP vs. BI-Y.

**Table 7:** Basic Lag Model Estimation Results for Disobedience (MLR Estimation), Unstandardized Estimates

	Model 1 (*n* = 294) BI-Y+BI-YP vs. STS		Model 2 (*n* = 198) BI-Y vs. STS		Model 3 (*n* = 195) BI-YP vs. STS		Model 4 (*n* = 195) BI-Y vs. BI-YP	

Variables	Estimate	S.E.	Estimate	S.E.	Estimate	S.E.	Estimate	S.E.
3-Month follow-up disobedience ON:								
Previous year disobedience	0.075^***^	0.021	0.069^**^	0.023	0.076^**^	0.026	0.074^*^	0.033
Gender					−0.150	0.081		
Lives with					0.241^*^	0.097		
Intervention	−0.066	0.080	−0.001	0.092	−0.145^+^	0.087	−0.143^+^	0.085
6-Month follow-up disobedience ON:								
3-Month follow-up disobedience	0.400^***^	0.088	0.382^***^	0.093	0.418^***^	0.102	0.410^**^	0.126
Age	−0.074^*^	0.031	−0.091^*^	0.042	−0.054	0.036		
Family stress/trauma			−0.065^**^	0.022				
Intervention	0.126^+^	0.074	0.093	0.090	0.145^+^	0.083	0.007	0.097
12-Month follow-up disobedience ON:								
6-Month follow-up disobedience	0.242^*^	0.111	0.325^*^	0.131	0.218	0.150	0.181	0.132
Intervention	−0.062	0.114	−0.080	0.129	−0.053	0.132	0.006	0.128
18-Month follow-up disobedience ON:								
12-Month follow-up disobedience	0.350	0.262	0.381	0.345	0.298	0.297	0.454	0.315
Age	−0.249^**^	0.088	−0.254^**^	0.086	−0.296^*^	0.115		
Family income level			0.154	0.110				
Intervention	−0.319	0.307	−0.343	0.316	−0.260	0.343	0.054	0.300
Baseline disobedience ON:								
Age	−0.167^*^	0.080	−0.172	0.096	−0.179	0.095	−0.178	0.106
Gender (1 = female)	−0.148	0.231	−0.215	0.318	0.165	0.297	−0.344	0.226
Family income level	0.126	0.085	0.119	0.110	0.212^*^	0.095	0.097	0.104
Lives with (1 = mother only)	−0.038	0.250	0.021	0.350	0.118	0.313	−0.123	0.267
Race (1 = African-American)	0.944^**^	0.283	1.093^**^	0.350	0.953^**^	0.341	0.761^*^	0.359
Baseline disobedience ON:								
Ethnicity (1 = Hispanic)	1.033^***^	0.281	1.247^**^	0.375	1.342^***^	0.354	0.466	0.291
Family stress/trauma	0.144	0.074	0.124	0.096	0.168	0.089	0.137	0.082
Sexual risk at baseline	0.055	0.107	0.102	0.134	−0.051	0.128	0.119	0.136
Delinquency at baseline	0.270^*^	0.129	0.337^*^	0.165	0.175	0.143	0.247	0.155
Marijuana use at baseline	0.068	0.105	0.044	0.141	0.144	0.135	0.039	0.112
Attitudes toward school at baseline	−0.019	0.040	−0.014	0.052	−0.001	0.048	−0.044	0.046
Intercepts:								
Baseline disobedience	2.047	1.197	2.197	1.456	1.511	1.385	2.649	1.593
3-Month disobedience	−0.753^***^	0.075	−0.741^***^	0.076	−0.781^***^	0.084	−0.748^***^	0.070
6-Month disobedience	0.582	0.472	1.019	0.624	0.302	0.543	−0.366^**^	0.125
12-Month disobedience	−0.334^**^	0.127	−0.279^*^	0.136	−0.351^*^	0.149	−0.436^***^	0.123
18-Month disobedience	4.617^**^	1.351	4.114^***^	1.336	5.273^**^	1.723	0.769^**^	0.291
Residual Variances:								
Baseline disobedience	3.320^***^	0.436	3.850^***^	0.565	3.240^***^	0.543	2.640^***^	0.381
3-Month disobedience	0.258^***^	0.025	0.287^***^	0.028	0.227^***^	0.030	0.225^***^	0.029
6-Month disobedience	0.212^***^	0.026	0.201^***^	0.029	0.186^***^	0.030	0.247^***^	0.035
12-Month disobedience	0.339^***^	0.030	0.331^***^	0.040	0.351^***^	0.039	0.331^***^	0.032
18-Month disobedience	1.347^***^	0.338	1.369^**^	0.426	1.384^**^	0.415	1.305^**^	0.422
*R^2^:*								
Baseline disobedience	0.116^*^	0.035	0.129^**^	0.045	0.131^**^	0.041	0.124^**^	0.044
3-Month disobedience	0.080^*^	0.040	0.067	0.045	0.176^**^	0.059	0.089	0.054
6-Month disobedience	0.213^**^	0.070	0.268^***^	0.069	0.231^*^	0.093	0.144	0.085
12-Month disobedience	0.045	0.040	0.081	0.063	0.032	0.042	0.028	0.040
18-Month disobedience	0.117	0.061	0.136	0.079	0.135	0.075	0.051	0.066

*Note*. Two-tailed *p*-values:

* *p* < .05

** *p* < .01

*** *p* < .001.

One-tailed significance levels (intervention effect):

+ *p* < .05

^++^*p* < .0125.

**Table 8:** Basic Lag Model Estimation Results for Inappropriate Behavior (WLSMV Estimation), Unstandardized Estimates

	Model 1 (*n* = 294) BI-Y+BI-YP vs. STS		Model 2 (*n* = 198) BI-Y vs. STS		Model 3 (*n* = 195) BI-YP vs. STS		Model 4 (*n* = 195) BI-Y vs. BI-YP	

Variables	Estimate	S.E.	Estimate	S.E.	Estimate	S.E.	Estimate	S.E.
3-Month follow-up inappropriate behavior ON:								
Previous year inappropriate behavior	0.786^***^	0.159	0.721^***^	0.186	0.746^***^	0.182	0.774^***^	0.172
Intervention	0.226	0.291	0.194	0.378	0.209	0.342	0.057	0.394
6-Month follow-up inappropriate behavior ON:								
3-Month follow-up inappropriate behavior	0.181^***^	0.041	0.164^***^	0.046	0.187^***^	0.042	0.190^***^	0.050
Intervention	0.032	0.086	0.130	0.104	−0.053	0.093	−0.156^+^	0.093
12-Month follow-up inappropriate behavior ON:								
6-Month follow-up inappropriate behavior	0.934^***^	0.232	0.900^***^	0.257	0.861^**^	0.290	0.754^***^	0.202
Intervention	−0.208^+^	0.125	−0.168	0.169	−0.227	0.157	0.001	0.152
18-Month follow-up inappropriate behavior ON:								
12-Month follow-up inappropriate behavior	0.266^**^	0.108	0.414^*^	0.185	0.239	0.124	0.133	0.087
Intervention	−0.134	0.122	−0.184	0.170	−0.013	0.219	−0.036	0.126
Baseline inappropriate behavior ON:								
Age	−0.150^***^	0.035	−0.140^**^	0.044	−0.143^***^	0.040	−0.156^**^	0.047
Gender (1 = female)	−0.347^***^	0.085	−0.341^**^	0.106	−0.324^**^	0.098	−0.360^**^	0.111
Family income level	−0.026	0.035	−0.051	0.043	−0.028	0.044	0.003	0.044
Lives with (1 = mother only)	0.075	0.082	0.120	0.112	0.031	0.094	0.086	0.108
Race (1 = African-American)	0.267^*^	0.111	0.198	0.138	0.303^*^	0.118	0.280	0.143
Ethnicity (1 = Hispanic)	0.044	0.091	0.055	0.113	0.084	0.100	−0.015	0.119
Family stress/trauma	−0.002	0.023	−0.012	0.028	0.008	0.027	0.007	0.028
Sexual risk at baseline	0.106^**^	0.037	0.110*	0.045	0.107^**^	0.041	0.102^*^	0.051
Delinquency at baseline	0.069	0.051	0.050	0.063	0.129^*^	0.063	0.031	0.064
Marijuana use at baseline	0.005	0.040	−0.053	0.053	0.042	0.047	0.010	0.052
Attitudes toward school at baseline	−0.023	0.014	−0.039^*^	0.017	0.003	0.015	−0.032	0.018
Intercepts:								
Baseline inappropriate behavior	1.837^**^	0.541	2.011^**^	0.702	1.492^*^	0.612	1.825^**^	0.678
6-Month inappropriate behavior	−0.876	0.578	−0.860	0.691	−0.903	0.763	−0.865	0.707
12-Month inappropriate behavior	3.417^***^	0.957	4.260^**^	1.244	2.579^*^	1.261	3.550^**^	1.131
18-Month inappropriate behavior	−0.610	0.746	−0.923	1.075	0.040	1.091	−1.050	0.866
Thresholds:								
3-Month inappropriate behavior$1	0.428	1.701	0.021	1.983	0.618	2.287	0.444	2.464
3-Month inappropriate behavior$2	1.285	1.685	0.931	1.952	1.770	2.347	1.079	2.445
3-Month inappropriate behavior$3	2.100	1.656	1.515	1.996			1.923	2.407
Residual Variances:								
Baseline inappropriate behavior	0.324^***^	0.053	0.334^***^	0.071	0.271^***^	0.053	0.356^***^	0.070
6-Month inappropriate behavior	0.117^***^	0.033	0.117^**^	0.035	0.095^**^	0.032	0.134^***^	0.037
12-Month inappropriate behavior	0.171^***^	0.044	0.145^**^	0.052	0.223^**^	0.066	0.153^***^	0.044
18-Month inappropriate behavior	0.171^***^	0.044	0.165^**^	0.048	0.173^***^	0.048	0.132^**^	0.043

	Estimate	Scale Factors	Estimate	Scale Factors	Estimate	Scale Factors	Estimate	Scale Factors
*R^2^:*								
Baseline inappropriate behavior	0.273		0.271		0.321		0.255	
3-Month inappropriate behavior	0.222	0.913	0.198	0.923	0.187	0.932	0.222	0.908
6-Month inappropriate behavior	0.269		0.254		0.311		0.279	
12-Month inappropriate behavior	0.456		0.458		0.344		0.409	
18-Month inappropriate behavior	0.143		0.250		0.103		0.038	

*Note*. Two-tailed p-values:

* *p* < .05

** *p* < .01

*** *p* < .001.

One-tailed significance levels (intervention effect):

+ *p* < .05

^++^*p* < .0125.

**Table 9:** Basic Lag Model Estimation Results for Skipping School (WLSMV Estimation), Unstandardized Estimates

	Model 1 (*n* = 294) BI-Y+BI-YP vs. STS		Model 2 (*n* = 198) BI-Y vs. STS		Model 3 (*n* = 195) BI-YP vs. STS		Model 4 (*n* = 195) BI-Y vs. BI-YP	

Variables	Estimate	S.E.	Estimate	S.E.	Estimate	S.E.	Estimate	S.E.
3-Month follow-up skipping school ON:								
Previous year skipping school	0.203^***^	0.051	0.216^**^	0.064	0.266^**^	0.077	0.191^**^	0.061
Intervention	0.205	0.320	0.232	0.379	0.214	0.412	0.036	0.298
6-Month follow-up skipping school ON:								
3-Month follow-up skipping school	0.403^**^	0.127	0.798^**^	0.271	0.288^*^	0.118	0.351^**^	0.122
Intervention	0.149	0.385	−0.295	0.692	0.368	0.464	0.517	0.509
12-Month follow-up skipping school ON:								
6-Month follow-up skipping school	0.178^**^	0.053	0.172^**^	0.052	0.204^**^	0.076	0.176^**^	0.058
Intervention	0.018	0.118	0.032	0.175	0.041	0.137	0.100	0.154
18-Month follow-up skipping school ON:								
12-Month follow-up skipping school	0.394	0.249	0.409	0.286	1.398^*^	0.682	0.852	0.637
Intervention	−0.062	0.550	0.101	0.753	−0.642	1.840	−0.917	0.861
Baseline skipping school ON:								
Age	−0.092	0.065	−0.069	0.083	−0.126	0.078	−0.070	0.091
Gender (1 = female)	−0.373^*^	0.150	−0.529^**^	0.192	−0.291	0.168	−0.303	0.207
Family income level	0.092	0.065	0.074	0.070	0.058	0.084	0.117	0.090
Lives with (1 = mother only)	−0.078	0.157	−0.191	0.202	−0.008	0.195	−0.023	0.216
Race (1 = African-American)	0.185	0.183	0.074	0.216	−0.008	0.246	0.452	0.244
Ethnicity (1 = Hispanic)	0.236	0.162	0.149	0.199	0.032	0.194	0.466^*^	0.225
Family stress/trauma	−0.009	0.038	0.002	0.049	−0.047	0.052	0.023	0.046
Sexual risk at baseline	0.143^*^	0.073	0.171^*^	0.081	0.077	0.083	0.176	0.105
Delinquency at baseline	−0.032	0.080	−0.078	0.087	0.081	0.122	−0.090	0.108
Marijuana use at baseline	0.049	0.079	−0.060	0.095	0.099	0.095	0.091	0.107
Attitudes toward school at baseline	−0.033	0.025	−0.040	0.029	0.002	0.034	−0.062	0.034
Intercepts:								
Baseline skipping school	1.634	0.880	1.577	1.116	2.154	1.120	1.084	1.240
18-Month skipping school	−0.142	0.666	−0.205	0.819	−0.352	0.678	0.734	0.980
Thresholds:								
3-Month skipping school$1	0.064	1.646	−0.838	2.164	0.704	2.233	0.630	2.266
3-Month skipping school$2	0.770	1.658	−0.142	2.194	1.342	2.228	1.466	2.274
3-Month skipping school$3	1.910	1.954	0.878	2.566	2.329	2.528		
6-Month skipping school$1	0.855	2.222	−2.757	3.636	2.977	2.832	0.581	3.530
6-Month skipping school$2	1.570	2.231	−1.824	3.555	3.814	2.822	1.226	3.478
6-Month skipping school$3	2.521	2.414			4.576	2.895	1.965	3.698
18-Month skipping school$1	−1.891	4.220	−1.344	6.755	3.382	12.385	−5.363	13.146
18-Month skipping school$2	−1.209	4.350	−0.463	7.331	4.207	12.404	−4.795	13.147
Residual Variances:								
Baseline skipping school	1.064^***^	0.088	1.029^***^	0.107	0.933^***^	0.114	1.146^***^	0.113
12-Month skipping school	0.177^***^	0.034	0.144^***^	0.030	0.117	0.060	0.168^***^	0.034

	Estimate	Scale Factors	Estimate	Scale Factors	Estimate	Scale Factors	Estimate	Scale Factors
*R^2^:*								
Baseline skipping school	0.089		0.120		0.074		0.135	
3-Month skipping school	0.055	0.979	0.064	0.977	0.077	0.696	0.046	0.980
6-Month skipping school	0.154	0.925	0.403	0.774	0.119	0.959	0.166	0.941
12-Month skipping school	0.176		0.257		0.298		0.209	
18-Month skipping school	0.033	0.984	0.034	0.984	0.270	0.872	0.225	0.934

*Note*. Two-tailed *p*-values:

* *p* < .05

** *p* < .01

*** *p* < .001.

One-tailed significance levels (intervention effect):

^+^*p* < .05

^++^*p* < .0125.

## Footnotes

1 We also planned to analyze Hillsborough County Schools data in school grades, promotion, suspensions, expulsions and placement in alternative schools. We were unable to do so for the following reasons. First, regarding school grades, we encountered a number of difficulties in attempting to examine the school grades data. The grades for each youth are listed by quarter; however, in some of the data files, the grades don’t specify which class they were received in; they are only assigned a course number and there is no label to determine what class it was. Further, some youth have blank cells where a grade should be; there is no code to determine whether this is because the youth was not in school, did not attend class, or what the case may be. As well, instead of a letter grade, some youth have a “P” listed in their cell instead of a letter grade which most likely means “passing” but (1) it is not so labeled in the data file and (2) is different from a letter grade of “A,” “B,” “C,” or “D” which can be quantified. Also, if a youth is assigned an “F” as a grade it doesn’t indicate whether that was a class that had a pass/fail system or the letter grade system. In addition to P/F, there are appear to be other things listed in the cells instead of a grade from time to time, such as “H” or “+” or “F*” or “U;” and there is no label to denote exactly what these symbols mean. In addition to the quarter grades, there is a column for “exam” and some youth have a grade listed while some don’t; this is another case of missing information without knowing why the information is not listed. Finally, while some youth have a blank cell in the grades section, still other youth have an “NG” which apparently means “no grade;” again, there is no label in the data to confirm that meaning. Some youth have a blank space while others have an NG, which doesn’t tell us why exactly a youth might not have a grade for that quarter. It is difficult to draw meaningful conclusions from such disparate data. Second, data on suspensions, and expulsions were available on a school year basis only (mid-August one year through mid-June the next year), which prevents meaningful analyses of these data. Third, school records indicated few study youth were placed in alternative schools (3-month follow-up: 0.3%, 6-month follow-up: 1.0%; 12-month follow-up: 2.0%; 18-month follow-up: 0.3%) or graduated (3-month follow-up: 2.7% 6-month follow-up: 0.3%; 12-month follow-up: 1.3%; 18-month follow-up: 2.0%), which also prevents meaningful analyses of these data. The school data base is basically an MIS system, designed to record school level events and transactions, not a research data base. The school problem behavior data we analyzed were the most research capable data on the school activities of the truant youth in our study we could examine.
